# British Medicinal Plants

**Published:** 1890-07

**Authors:** Alfred Heath

**Affiliations:** London, England


### BRITISH MEDICINAL PLANTS.

ALFRED HEATH, M. D., F. L. S., LONDON, ENGLAND.

### RANUNCULACEÆ (CONTINUED).

*Ranunculus sceleratus* (the Celery-leaved Crowfoot, Marsh Crowfoot), found in damp places and borders of ditches, but also growing in the water. The leaves of this plant are so very acrid that the beggars in Switzerland are said to produce very fœtid and acrimonious ulcers by rubbing their legs with them. They could not well have selected a better medicine in a homœopathic sense, as one of its chief symptoms, when taken by the healthy, is *to produce laziness and want of disposition to perform any mental labor*. It is to be hoped that after the healing of their legs they may cease to be beggars. It would be interesting to know if the homœopathicity of the drug cured their laziness. There is a good proving of this drug in Jahr's *Materia Medica.* It produces many kinds of headache. Vomiting, sweetish taste in the mouth, profuse lachrymation, smarting in the eyes, earache of the right ear, smarting in the nose and excessive secretion of watery mucus, sneezing, toothache, and a great many other symptoms. Amongst others it is especially useful for *corns* with intolerable *burning*, stitches, pricking, sticking, boring pains along the whole sole of the right foot; itching and furious smarting of the soles, *sudden stitches in the forepart of the right big toe, as if a needle were thrust in deep,* recurring at short intervals, sometimes passing into a burning.

*Ranunculus Flammula* (Lesser Spearwort).—Small water crowfoot, also found in wet places. Taste very acrid and hot, a small quantity of the herb eaten produces vomiting, spasms of the stomach, and delirium. Applied externally, it vesicates the skin. According to Jahr it produced inflammation and gangrene of the arms down to the tendons and bone, in the case of a woman, from applying the plant to the wrist. It produces in horses on eating it, excessive distention of abdomen and inflammation and gangrene of the abdominal organs, but it is said to promote digestion when a very small quantity is eaten.

*Ranunculus Ficaria* (Pile-wort. Lesser Celandine).—This plant has been deemed anti-scorbutic, and the root was esteemed as a specific for piles, hence its common name. This is one of the earliest flowers we have, and its shining green leaves and beautiful golden flowers are seen by the side of almost every water-course and ditch in the early spring. Besides its use for painful and bleeding piles, swelling, etc., it is said to have been used with considerable success for external wounds and bruises and spitting of blood. An ointment is made by boiling the bruised leaves with lard, which is said to be very healing. No regular proving of the plant exists.

*Ranunculus repens* (Creeping Crowfoot), very common, found in meadows and pastures, also very acrid, producing smarting of the eyes, profuse lachrymation, and curious dreams; he fancies, while yet awake, that he is in a large city, and sees well-dressed people, masquerades, Turks, etc. I, myself, had a very curious dream or vision, while awake, some years ago, just as I was recovering from an attack of typhoid fever; for *three nights in succession, immediately on closing my eyes, and while perfectly awake*, I imagined I was standing on the brink of the most profound abyss. The horror caused me instantly to open my eyes, but there was no return of the vision when I closed them again until the next night, when the same thing occurred, it also disappearing on opening my eyes. On the next night it again occurred. It is possible I may have been under the influence of the same drug, or one producing similar symptoms, as I was in the country botanizing at the time, and in a part where this plant grows, but I had no knowledge of such being the case. I have no doubt the drug would cure this condition of things—namely, day-dreams. A proving of it would show what its virtues are. Evidently the Ranunculus family produce a good deal of mental languor, and should be especially thought of for sleepy, inactive people, and persons subject to day-dreams, etc. The following were the effects on a flock of sheep: Several fell down as if struck by lightning. The eyes rolled, the breathing was hurried and aggravated; some reeled and died with their *heads bent toward the left groin*. The mucous membrane of the eyes was injected; the mouth dry, the abdomen was slightly distended, rumination ceased; some of them raised themselves, reeled, fell down again, bleated pitifully; most were in profound coma. Sulphuric Ether in milk gave much relief, but great weakness of the feet remained behind.

*Ranunculus acris* (Upright Crowfoot. Meadow Crowfoot).—This, as its name implies, is of a very acrid nature, and was at one time employed externally as a vesicatory. It produces a quicker effect than an ordinary blistering plaster, but it also produces ulcers that are very difficult to heal; therefore it has been used (allopathically) where long-continued topical stimulus is required to produce discharge from the part, as in an issue. The homœopathic materia medica gives no proving of this plant, but only records some of its poisonous symptoms. Applied to the temple it produces headache, intolerable heat and fainting. Applied to the joints, it produces soreness of the joints, and obstinate ulcers as far as the knees; both feet looked burnt; red, hot blisters appeared here and there; several places became gangrenous on the third day, and trembling and fainting occurred. Wounds of twenty years' standing began to improve after applying a decoction of the leaves to the legs.

*Ranunculus bulbosus* (Bulbous-rooted Crowfoot) common in meadows and pastures in May, easily known by its *reflexed calyx* and bulbous root, the only buttercup at this time in pastures with a reflexed calyx; so that finding this, one is sure there is a bulb at the bottom of the stem. A curious fact concerning this plant is that the new bulb for the next year is always a little higher up toward the surface than the old one. What takes place the following year I do not know; if they went on doing this for a year or two they would be out of the ground; perhaps the rooting process draws them down again. It is the plant commonly called the buttercup, and many think that it is on account of the yellow color of these flowers that the butter receives its yellow color in the spring, whereas the cows will not eat any of the crowfoot while green on account of its hot, caustic taste. This plant was said to be a remedy against the plague. The homœopathic materia medica gives a long proving of it. It has many symptoms similar to *Ranunculus sceleratus*, but whereas the latter produces a lazy feeling, the former produces a *dread* of labor; it also causes many other mental symptoms, *ill-humor and disposition to quarrel and scold*, fear of being alone, afraid he will be haunted by ghosts. It is also most intensely acrid, merely bruising the roots is sufficient to cause the nose and eyes to stream with water, so that one can scarcely see, smarting and soreness of the eyes, and a host of other disagreeable symptoms. Some years ago I had occasion to bruise and prepare some of these roots. At the time I had a heavy cold in my head. The punishment I received I shall never forget. It seemed as though a fountain had opened in my head. I could not see from the flow of water from my eyes. The nose also streamed with water, but in the morning every vestige of the cold I previously had was gone. This incident shows how true is the "law of similars"—that like will cure like.

This finishes the *Ranunculus* genus as far as they have been used in Homœopathy. The next in order is the tribe Helleboreæ.

*Caltha palustris* (Marsh Marigold), found in marshy places, water-courses, and damp meadows. Its large, golden-yellow flowers almost as large as the Christmas rose (*Helleborus niger*), are well worth gathering in the spring, and make a very handsome bouquet for the drawing-room, with a few only of their shining green leaves, and they last a long time in water. The young buds of this plant, when properly pickled, are said to be a very good substitute for capers. There is some account of this plant given in the *British Journal of Homœopathy*, Vol. II.

